# The effect of nebivolol on erectile function in the cases with coronary artery bypass surgery: A protocol for a systematic review and meta-analysis of randomized controlled trials

**DOI:** 10.1097/MD.0000000000021588

**Published:** 2020-08-07

**Authors:** Yali Yang, Shanshan Yong, Fuhao Li, Liang Dong, Degui Chang

**Affiliations:** a Hospital of Chengdu University of Traditional Chinese Medicine; b Department of Andrology, The Reproductive and Women-Children Hospital, Chengdu University of Traditional Chinese Medicine, Chengdu, Sichuan Province, China.

**Keywords:** coronary artery bypass, erectile dysfunction, IIEF-5, nebivolol, systematic review

## Abstract

**Background:**

Erectile dysfunction is a common disease. It affects the quality of life of both husband and wife and its prevalence is higher in patients with overt cardiovascular disease or cardiovascular risk factors. In recent years, multiple studies confirm that nebivolol exerts protective effects on erectile function against the disruptive effects of cardiopulmonary bypass in patients undergoing coronary artery bypass grafting, but its quality and efficacy have not been systematically evaluated. Therefore, it is necessary to carry out a systematic review and meta-analysis to fully evaluate the efficacy and safety of nebivolol on erectile function in the cases with coronary artery bypass grafting.

**Methods and analysis:**

Chinese and English literature of nebivolol on erectile function in the cases with coronary artery bypass surgery published before August 31, 2020 will be comprehensive searched in PubMed, Cochrane Library, EMBASE, WANFANG, China National Knowledge Infrastructure, VIP Chinese Science and Technology Journal Database, Chinese biomedical document service system, and Clinicaltrials.gov. Only randomized controlled trials that meet the eligibility criteria will be included. Two researchers will independently complete literature screening, data extraction and assess the risk of bias, and the third investigator will handle disagreements. Our main evaluation includes 2 outcome indicators including the international index of erectile function 5 score and adverse events. RevMan 5.3 and Stata 14.0 will be used to conduct this systematic review. The preferred reporting items for systematic reviews and meta-analysis protocols (PRISMA-P) statement is followed in this protocol and the PRISMA statement will be followed in the completed systematic review.

**Conclusion and dissemination:**

The efficacy and safety of nebivolol on erectile function in the cases with coronary artery bypass grafting will be evaluated. We will publish the results of this systematic review in peer-reviewed journals to provide new evidence to clinicians.

**Ethics and dissemination:**

Ethical approval is not required as the review is a secondary study based on published literature. The results will be published in a public issue journal to provide evidence-based medical evidence for urologists and andrologists to make better clinical decisions.

**Registration information:**

INPLASY202060110.

## 1 Introduction

Erectile dysfunction (ED) refers to the inability of men to obtain and maintain an adequate penile erection to maintain satisfactory sexual intercourse, and it is one of the most common male sexual dysfunctions.^[1]^ During the last 2 decades, significant advances in the pathophysiology of erectile dysfunction revealed that erectile dysfunction is of vascular origin in the majority of cases, and thus its prevalence is higher in patients with overt cardiovascular disease or cardiovascular risk factors.^[2]^

Ischemic cardiac diseases are common health problems for the elderly population and despite the new treatment methods, coronary artery bypass grafting (CABG) is still commonly performed. CABG with the use of cardiopulmonary bypass (CPB) may cause endothelial dysfunction by reducing the synthesis and release of plasma nitric oxide (NO), Endothelial dysfunction is a major cause of ED.^[3]^ Myocardial revascularization is used to increase supply part of “the supply/demand ratio” in myocardial ischemia, on the other hand, decreasing the demand part of “the supply/demand ratio” is also very important. β-blockers are drugs used as anti-ischemic, antihypertensive, and antiarrhythmic agents to reduce extra unnecessary energy consumption. In the cases with CABG operation and ischemic cardiac disease, it is the preferred treatment method. When choosing the β-blocker, its side effects must be considered together with the side effects of CPB, it is reported that these agents indicated as class I use in CABG operations cause ED.^[4,5]^

It is observed that the third-generation β-blockers not only have beta adrenoreceptor-blocking activity but have additional vasodilating properties.^[6]^ The third-generation β-blocker agent nebivolol that has a high β1-adrenoceptor selectivity leads directly to arterial and venous vasodilatation by NO system activation. Given this feature of nebivolol, in contrast to other β-blocker agents, it may increase NO release and improve erection or may not cause impotence.^[7]^

In recent years, the clinical trials of nebivolol on erectile function in the cases with coronary artery bypass grafting have increased, multiple studies confirm that nebivolol exerts protective effects on erectile function against the disruptive effects of CPB in patients undergoing CABG. But its quality and efficacy have not been systematically evaluated. Therefore, it is necessary to carry out a systematic review and meta-analysis to fully evaluate the efficacy and safety of nebivolol on erectile function in the cases with coronary artery bypass grafting.

## 2 Review objectives

The purpose of this systematic review is to evaluate the efficacy of nebivolol for erectile function in the cases with coronary artery bypass surgery men, provide evidence-based medical evidence, and provide better clinical decisions in the future.

## 3 Methods

### 3.1 Protocol and registration

The protocol of this systematic review will refer to the statement of Preferred Reporting Items for Systematic Review and Meta-Analysis Protocols (PRISMA-P) checklist.^[8,9]^ This protocol has been registered on the International Platform of Registered Systematic Review and Meta-analysis Protocols (registration number: INPLASY202060110) which could be available at https://inplasy.com/.

### 3.2 Eligibility criteria

We list the inclusion and exclusion criteria below.

#### 3.2.1 Types of studies

Only include randomized controlled trials (RCTs) that meet eligibility criteria. All the case reports, patient series, retrospective studies, self-controlled or before and after controlled studies, animal studies, reviews, laboratory researches, observational studies, meta-analyses, letters, and other second-hand studies will be excluded.

#### 3.2.2 Participants

##### 3.2.2.1 Included population

Male patients after diagnosis of coronary artery disease and who are referred to coronary artery bypass, and having a regular sexual partner. Regardless of whether they had ED, no limit with age. All medications used in the preoperative or postoperative period were recorded.

##### 3.2.2.2 Excluded population

The patients with hormonal ED were excluded, previous genitourinary or prostate surgery that might affect sexual activity, neurological disease, major depression, hypothalamo-hypophyseal axis hormone abnormality, peripheral artery disease (Ankle brachial index: <0.9), renal failure (creatinine: >1.5 mg/dL), and diabetes mellitus.

#### 3.2.3 Interventions

This group was treated with nebivolol, a comparison of nebivolol against other drugs will be included, limited to RCTs for drug therapy. If nebivolol is used as a control in the trial, and the other drug is an intervention, then we will consider reversing the order of these 2 interventions in this systematic review, that is, nebivolol will be considered an intervention, another drug is a control measure.

#### 3.2.4 Control measures

The control group receive other adrenoceptor β-blockers, such as “metoprol” or other drugs.

#### 3.2.5 Outcomes

##### 3.2.5.1 Primary outcome

IIEF-5 score.

##### 3.2.5.2 Secondary outcomes

Adverse events: all adverse events reported in the included studies.

### 3.3 Search strategy

#### 3.3.1 Information sources

English literature in Cochrane library, EMBASE, PubMed, and Chinese literature in China National Knowledge Infrastructure (CNKI), Chinese biomedical document service system (SinoMed), VIP Chinese Science and Technology Journal Database (VIP), WANFANG data will be included. Related RCTs will be collected and selected before August 31, 2020. The searching work will be done in September 2020 and updated before the systematic review has completed.

Subject heading, free text words will be used to search in Cochrane library, EMBASE, PubMed. In Cochrane library and EMBASE, the use of free words will be limited within title, abstract, and keywords, but in PubMed, limited in title/abstract. The “topic” field will be used for the search of CNKI and WANFANG, and the “title or keyword” filed for the search of VIP. The subject heading plus free words form will be used to retrieve SinoMed.

Medical Subject Heading or text key words “Nebivolol” AND “Erectile dysfunction” or “ED” AND “coronary artery bypass.” Chinese form of the above terms will be used in Chinese search. A specific search example for PubMed is shown in Table 1.

**Table 1:**
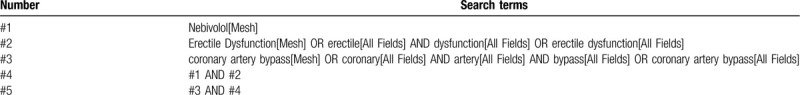
Example of Pubmed search strategy.

#### 3.3.2 Other sources of search

We will scan Clinicaltrials.gov for registered clinical trials. Besides, we will also scan the Baidu's academic search engine, and database of Chengdu University of Traditional Chinese Medicine Library, dissertations of degrees will be included.

### 3.4 Selection of studies

Document management will be conducted by Endnote X8 software. The software will be used to filter duplicate documents first, then delete repeated literature by reading titles, abstracts, and other relevant information.

**Figure 1:**
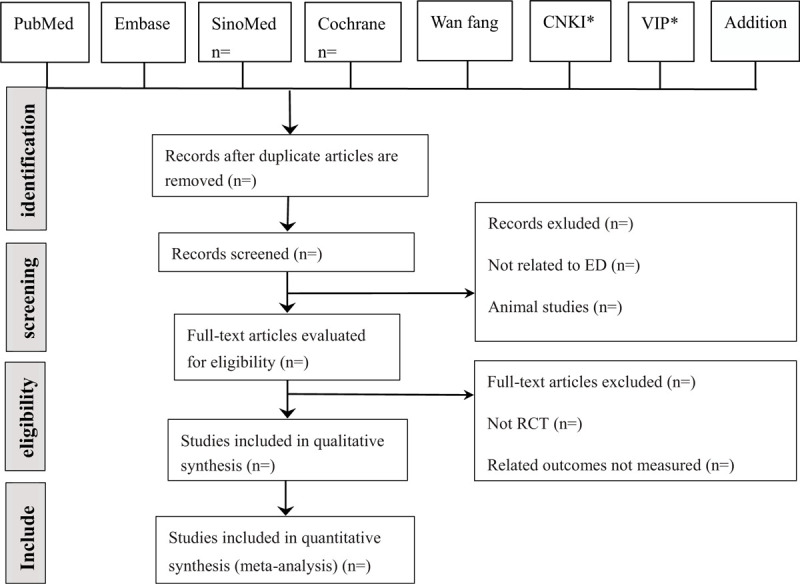
Study selection flow chart.

Studies will be removed if they don’t meet the inclusion criteria. Then the literature will be further screened. Two authors (YY, SY) will conduct the further detailed screening and data extraction of the documents, the controversial areas will be resolved through discussions with another member (FL). If 2 or more articles have repeated or staged research results, only the articles with the largest sample size, the most complete intervention, and follow-up time are included. When the team member is unable to judge the duplication, the original research author will be contacted by email for more details of the study. A flow diagram of the study selection is shown in Fig. 1.

### 3.5 Data extraction and management

The review group will discuss the information and a data extraction form will be produced before the formal process of data extraction. The 2 independent reviewers (YY, SY) will extract data from the included study, the data in 3 to 4 articles will be extracted in advance to test the consistency of data extraction and the accuracy of the table, all disagreements will be discussed with the third member (FL). The content of data extraction is as follows.

1. General characteristics: the name of first author, publishing year, article title, location, time of study, email.
2. Design and methodology of studies: study design, sample size, randomized information, allocation concealment, blind method, diagnostic criteria, outcome indicators, safety indicators, statistical methods.
3. Participants/patients: age, body mass index, ejection fraction, cross-clamp time, cardiopulmonary bypass time, proportion of hypertension, days in intensive care unit, days in hospital, proportion of dysrhythmia, proportion of postoperative myocardial infarction, baseline level.
4. Information of Exposure and control: name of drug, method of administration, dosage and frequency, and duration, combination medicine.
5. Outcome indicators: IIEF-5 scores at different time points; data of adverse events and specific information.
6. Risk of bias: random sequence generation, allocation concealment, blind of researchers and patients, complete of data, selective reporting.
7. Other study information: conflict of interest, funding situation.

### 3.6 Risk of bias assessment

Two reviewers (YY, SY) will independently use different scales to assess the risk of bias based on the extracted data information: Selection bias, performance bias, detection bias, attrition bias, reporting bias, and other bias, with the use of Cochrane Collaboration Network Risk Assessment Tool.^[10]^ Inconsistencies between review authors on the risk of bias will be discussed and resolved with the third author (FL). Assessment items include information of random sequence generation, assignment hiding, blind of researchers and patients, data integrity, selective reporting, and other bias.^[10]^ Each item of bias situation includes high risk, unclear, and low risk.^[10]^ The risk of bias will be presented as chart by the use of Review Manager 5.3 software.^[10]^

### 3.7 Data analysis and synthesis

Descriptive analysis or narrative synthesis will be performed if the clinical heterogeneity among the trials is significant or when the data cannot be synthesized or results data cannot be extracted. Only included trials are clinically homogeneous, and the data are sufficiently similar and homogeneous, a meta-analysis will be performed.^[11,12]^ Chi-square tests will be used to test the heterogeneity and I2 statistic will be used to test the size of heterogeneity.^[11,12]^ We will use Cochran's Q statistic and I2 statistic to test heterogeneity. *P* < .10 is heterogeneous or I2 >50% will be considered the heterogeneity is significant. A fixed effect model (Mantel–Haenzel method for risk ratio and Inverse Variance for mean difference) will be applied in the case of I2 < 50%. When I2 > 50%, we will analyze the heterogeneity causes: age, the severity of the disease, the Interventions’ dose and length, then conduct subgroup analysis.^[11,12]^ If the heterogeneity is still significant after sensitivity analysis and subgroup analysis, we will use random effects model (D-L method).^[11]^ The effect size will be measured by Z test, and the *P* value ≤.05 will be considered statistically significant.^[11,12]^ Mata-analysis will be presented as forest plots by the software RevMan 5.3.

### 3.8 Subgroup analysis

Subgroup analysis will be performed according to age, body mass index, blood pressure, baseline level, different time point of outcome measurement.

### 3.9 Sensitivity analysis

Sensitivity analysis will be used to test the stability and reliability of meta-analysis. It can be done by eliminating each study individually or using random-effect model (DerSimonian & Laird method) to test the results after using the fixed effect model.^[11,12]^

### 3.10 Publication bias

If over 10 studies are available, Egger test (by Stata software 14.0) and funnel plot will be used to reveal potential publication bias.

### 3.11 Grading the quality of evidence

We will use GRADE tool^[13]^ to assess the quality of evidence. According to 5 key domains: risk of bias, consistency, directness, accuracy, and publication bias, the level of evidence for each outcome can be divided into high quality, moderate quality, low quality, and very low quality levels.^[14]^

## 4 Discussion

β-blocker agents are particularly preferred in the treatment of hypertension and cardiovascular diseases due to their antihypertensive, antiarrhythmic, and anti-ischemic properties. For this reason, the use of β-blockers after CABG surgery is common and one of the important side effects is the impact on one's sexual life.^[15]^ Several mechanisms are mentioned to explain it, among which the decrease of sympathetic tone is particularly obvious because β-blockers are noncardioselective, vasodilation in the cavernous body is hindered, and the effect of luteinizing hormone reduces testosteroneemia or sleepiness or depression caused by β-blockers leads to decreased libido.^[16]^ Caused by autonomic innervation of the penis, NO acts as a local neurotransmitter of noncholinergic nonadrenergic nerves. NO leads to increased intracellular cyclic guanosine monophosphate accumulation, which causes corporeal smooth muscle relaxation. The role of NO in the physiology of male sexual function has been shown as the primary modulator of penile erection. So, drugs resulting in NO release may improve erectile function.^[15,17]^

Nebivolol is one of the third-generation β-blocking agents developed in recent years, it was developed and patented in the 1980s and came into medical use in Europe in 1997,^[18]^ compared with other β-blockers, there was no significant difference in terms of postoperative anti-ischemic effects.^[19]^ Nebivolol exhibits highly selective β1-AR blockade and has vasodilatory properties secondary to the enhancement of nitric oxide bioavailability.^[20]^ Experimental data demonstrate that nebivolol results in increases in endothelial nitric oxide synthase activation and phosphorylation, and endothelium-dependent relaxation of the corpora cavernosa,^[21]^ which exerts protective effects on erectile function against the disruptive effects of CPB in patients undergoing CABG.

There are some pieces of evidence based on RCTs for the efficacy of nebivolol on erectile function in the cases with coronary artery bypass grafting, but no relevant systematic review. Therefore, we will make a systematic review to provide evidence-based medical evidence for the clinical use of nebivolol. It will also provide recommendations for further research in the future.

This systematic review uses the IIEF-5 questionnaire score as an outcome indicator. The questionnaire has 5 questions, including erection confidence, erection hardness, maintenance of erection, the persistence of erection, and the satisfaction of sexual intercourse. The IIEF-5 score (a total of 25 points) is divided into normal erectile function (≥22 points); mild ED (12–21 points); moderate ED (8–11 points); severe ED (<8 points). Although this questionnaire is subjective, it has been verified, generally acknowledged, and has become an important tool for evaluating ED.^[22–25]^

We recognize that this study has some limitations. First, there may not be enough large samples of RCTs. Second, the quality of some RCTs may not be high and will affect the overall quality of the evidence. Therefore, we hope there will be more large-scale, multicenter, high-quality RCTs providing high-quality evidence in the future.

## Author contributions

**Conceptualization:** Yali Yang, Shanshan Yong, Degui Chang

**Data curation:** Yali Yang, Shanshan Yong, Fuhao Li

**Formal analysis:** Yali Yang, Shanshan Yong, Fuhao Li

**Funding acquisition:** Shanshan Yong, Liang Dong

**Investigation:** Yali Yang, Shanshan Yong, Fuhao Li, Degui Chang

**Methodology:** Yali Yang, Liang Dong

**Project administration:** Yali Yang, Shanshan Yong, Liang Dong, Degui Chang

**Resources:** Liang Dong, Degui Chang

**Software:** Yali Yang, Shanshan Yong, Fuhao Li

**Supervision:** Yali Yang, Shanshan Yong, Fuhao Li, Degui Chang

**Validation:** Yali Yang, Liang Dong, Degui Chang

**Writing – original draft:** Yali Yang, Shanshan Yong

**Writing – review & editing:** Fuhao Li, Liang Dong, Degui Chang
